# Consulting With First Nations Communities to Develop Text‐Based Support for Grieving Fathers

**DOI:** 10.1111/hex.70450

**Published:** 2025-09-29

**Authors:** Craig Hammond, Mick Adams, Richard Fletcher

**Affiliations:** ^1^ School of Health Sciences, College of Health, Medicine, and Wellbeing The University of Newcastle Newcastle Australia

**Keywords:** Aboriginal and Torres Strait Islander, co‐design, community consultation, digital SMS programme, Indigenous fathers, neonatal death, pregnancy loss

## Abstract

**Background:**

The loss occasioned through miscarriage, stillbirth or neonatal death is recognised as a traumatic event causing grief and sorrow in fathers. While the rate of Indigenous perinatal deaths is almost twice that of non‐Indigenous, there is little support available for Aboriginal and Torres Strait Islander grieving fathers.

**Objective:**

The SMS4DeadlyDads team partnered with Red Nose, the national charity supporting grieving parents, to co‐design text‐based support for grieving fathers with community representatives and clinicians.

**Design:**

A 2‐year consultation process with Indigenous services and stakeholders took place in urban and remote locations in Australia. The support for fathers following perinatal loss was assessed, and messages were adapted from those for non‐Indigenous fathers and evaluated. Final messages were reviewed by Red Nose clinicians for optimal delivery timing.

**Results:**

Community representatives noted the lack of support for new fathers. The culturally appropriate SMS4Deadlydads service delivering text messages to new fathers' mobile phones was welcomed as ‘something for dads’ and the potential to provide confidential support in cases of perinatal loss was recognised. The resulting set of messages was acceptable to indigenous and non‐Indigenous stakeholders.

**Conclusions:**

The successful development of the messages for Indigenous fathers demonstrates that respectful consultation led by experienced Indigenous leaders can ensure cultural safety and gain community commitment to address highly sensitive topics.

**Public Contribution:**

Indigenous community representatives and stakeholder service were involved in deciding on the value of the text messaging approach to fathers' grief, the identification of message topics, the wording used in the texts and the linked resources.

## 1 Introduction

The loss occasioned through miscarriage, stillbirth or neonatal death is recognised as a traumatic event causing grief and sorrow in mothers and fathers. The consequences for both parents can include depression and anxiety, post‐traumatic stress, and suicidal ideation, which may persist long after the miscarriage or death [1]. The stigma surrounding perinatal deaths can make conversations difficult for family and friends who may wish to offer support. Parents may be reluctant to engage in discussion of their experience, recognised as an important step in managing their grief, because they believe it will make others feel uncomfortable [2].

While social stigma will affect both parents, fathers' management of their grief may be incongruent with that of their partners. Fathers are more likely to increase their substance use and use goal‐oriented tasks as a coping strategy. They may suppress their emotional reactions to fulfil their role as strong support for the mother [3]. The focus of professional and community support on mothers' needs following perinatal loss can compound grieving fathers' isolation, leaving them with little access to effective support [4].

Colonisation has damaged Aboriginal men's identity through removal and incarceration, restricting their ability to pass on their culture [5]. Therefore, Aboriginal and Torres Strait Islander fathers, who are almost twice as likely as non‐Indigenous fathers to be confronted by the loss of their baby, may face additional challenges in managing their grief. The undermining of their role in the family and their connection to other male community members based on shared caring for the country has meant that grieving fathers may have few culturally safe services to turn to and may be isolated from community support [6]. As an Aboriginal father who had experienced perinatal loss told a consultation on community attitudes ‘When things are tough, we have to stay strong and if that means there is a stillbirth, we need to support our women, as well as somehow keep carrying on, even in our own grief. Sometimes we have our own traumas, and we are treated badly by the hospital staff, it makes us shame job. We don't need to carry shame; we are doing our best and we need support too’ [7, p. 7].

While digital programmes of support after perinatal loss have been designed for fathers [8] and digital psychotherapeutic interventions for grieving mothers have been found efficacious [9], no examples of digital programmes for Indigenous parents could be located. An important consideration when addressing perinatal loss for Aboriginal and Torres Strait Islander fathers is the social context and the cultural practices surrounding death and grieving. Aboriginal and Torres Strait Islander families are found in major cities with access to extensive health facilities and in remote communities with minimal health services. While successive national governments have attempted to address the gap in health outcomes [10], the age‐standardised mortality rate among Aboriginal and Torres Strait Islander populations is 1.7 times higher than for the non‐Indigenous population [11]. Health services in Australia have attempted to recognise the cultural protocols and responsibilities surrounding death, captured in the term Sorry Business, and brief explanations are included in leaflets and publications while recognising that practices vary widely [12, 13]. As well, sorry business is recognised as overlapping with cultural protocols of ‘men's business’ and ‘women's business’ understandings, which also vary across regions [14, 15].

The Healing Through Community (HTC) Grieving Fathers Project: In 2023, the Australian psychoeducation service SMS4DeadlyDads was contracted by Red Nose Australia to co‐design messages for Aboriginal and Torres Strait Islander fathers following pregnancy loss or stillbirth. The term ‘deadly’ signifies ‘very good’ in Aboriginal communities. SMS4DeadlyDads is an adaptation of SMS4dads (www.sms4dads.com) delivering text messages to fathers from early in the pregnancy until 1 year post birth. Aboriginal and Torres Strait Islander fathers enrol via the website with a minimal set of demographic questions and receive 3–6 brief (160‐character) texts per week. Since 2021, the programme has been funded by the Department of Health to offer the service nationally, and over 700 Aboriginal and Torres Strait Islander fathers have enrolled. The SMS4DeadlyDads team brought the experience of developing this national digital service for these fathers to the task of creating supportive resources for grieving fathers. It was recognised that, as fathers may enrol during the pregnancy, a number would experience pregnancy loss, stillbirth or neonatal death of an infant while receiving text messages from SMS4DeadlyDads. A previous collaboration with Red Nose had produced messages for mainstream grieving fathers [8].

The need to consult with Aboriginal and Torres Strait Islander communities is included as a matter of course in health service policies and publications. However, the mechanisms of engagement are rarely documented. In this paper, the consultation process undertaken by the SMS4DeadlyDads team in the HTC project is described to encourage the development of culturally appropriate supports for Aboriginal and Torres Strait Islander fathers and their families.

## 2 Materials and Methods

### 2.1 A Model of Consultation

In approaching Australian Aboriginal and Torres Strait Islander communities to conduct research, there are established guidelines to ensure the cultural safety of those involved. The National Health and Medical Research Council guidelines include six core values which must be taken into account: spirit and integrity, cultural continuity, equity, reciprocity, respect, and responsibility and the Aboriginal Health and Medical Research Council of NSW lists five key principles: net benefits for Aboriginal people and communities, Aboriginal community control of research, cultural sensitivity, reimbursement of costs and enhancing Aboriginal skills and knowledge [16, 17].

The development of messages with and for grieving Aboriginal and Torres Strait Islander fathers also required particular attention to the context of intergenerational trauma and the barriers to males accessing health services [18, 19]. Investigations into complex trauma assessment conducted as part of the Healing the Past by Nurturing the Future (HPNF) project have provided guidelines relevant to the task of discussing perinatal loss with community members [20]. The Alice Springs HPNF workshop involving 25 institutions identified six critical overarching themes: ensuring emotional and cultural safety; establishing relationships and trust; having capacity to respond appropriately and access support; incorporating less direct cultural communication methods (e.g., yarning and dadirri); using strengths‐based approaches and offering choices to empower parents; and showing respect, caring and compassion. The processes developed to inquire about Aboriginal men's sexual health and young Aboriginal fathers' health are also relevant to researching grieving fathers. These successful approaches commenced with trust‐building contact at the organisation level and utilised local Aboriginal and Torres Strait Islander men to reach and engage individual fathers [18, 21] (see Table 1). Ethical approval was received from the University of Newcastle Human Research Ethics Committee (H‐2022‐0016) and from the Aboriginal Health and Medical Research Council (1874/21).

**Table 1:** Model of consultation.

Phase 1		
Initial contact	Recruitment	Approach
Visit by the team to Aboriginal Community Controlled organisations and stakeholders	Using contacts from previous work‐together approaches are made to organisations	Introducing team members and explaining the HTC Grieving Fathers project and its relation to SMS4DeadlyDads. Key questions: What support is available for dads in the perinatal period? Would a text messaging service be useful for these fathers?

Phase 2		
Workshop	Recruitment	Approach
Discuss existing support for fathers and consider draft texts for grieving fathers	Contacts from Phase 1 and other stakeholders invited to attend in person	Introductions from participant services and explaining the HTC Grieving Fathers project and its relation to SMS4DeadlyDads. Hear from existing men's group leaders. Consider a small number of draft texts from the existing mainstream SMS4dads service.

Phase 3a		
Activity	Recruitment	Role
Advisory Group of men/fathers established in QLD, NSW and SA	Representatives from Aboriginal or Torres Strait Islander Health organisations	Provide advice on message development and appropriate resources for grieving fathers.

Phase 3b		
Workshop/Email	Recruitment	Approach
Draft messages considered in workshop format or sent to individuals to rate.	Contacts from Phases 1 and 2 and other stakeholders invited by leaflet or email to attend in person or provide feedback on draft texts	Consistent with previous co‐design development of text materials ratings on draft texts (e.g., easy to understand, culturally safe, suitable for recent pregnancy loss or stillbirth) are tabulated, and those with clearly negative comments are removed. Remaining texts are edited following suggestions [8, 22, 23]
Edited messages considered in workshop format or sent to individuals to review as appropriate for their communities	Those involved in the previous evaluation were recontacted to review the edited version	Comments on edited texts are reviewed, and texts with negative evaluative comments are removed. Remaining texts are edited following suggestions. In cases of disagreement, Advisory Group members were contacted to provide advice.
Final draft messages considered by experienced grief counsellors to advise on the timing of message delivery	Staff from Red Nose grief and loss service	Staff who had been briefed on the development of the messages asked to suggest the order and timing of message delivery

Phase 4	Recruitment	Approach
Resources for fathers to be linked to text messages drafted	Advisory Groups from QLD, NSW and SA	Draft resources reviewed by Advisory Group

Phase 5	Recruitment	Approach
Messages available for grieving fathers. Project results communicated to community organisations	Advisory Groups from QLD, NSW and SA and stakeholders	Advisory Group advise on materials and distribution of project results to communities.

### 2.2 Initial Consultations

Initial meetings in Cairns, Thursday Island, Adelaide and Port Augusta were arranged through personal contacts from previous work with these communities over many years. The SMS4DeadlyDads team, Indigenous leads Dr Mick Adams and Craig Hammond, and the Communications Officer Louie Hahn and Research Lead Richard Fletcher met with community representatives and perinatal health services supporting Aboriginal and Torres Strait Islander communities. In Cairns and Thursday Island, Skye Stewart (Indigenous lead Red Nose) accompanied the team. These meetings aimed to establish the existing level of support for new fathers, to explain the possible role of the SMS4DeadlyDads messages and to seek community involvement in co‐designing messages for grieving fathers.

Following the initial meetings with communities, workshops and meetings were held in Queensland (Cairns and Mareeba), New South Wales (Newcastle) and South Australia (Adelaide and Port Augusta).

To ensure local guidance in the creation of text messaging for grieving fathers, Aboriginal and Torres Strait Islander representatives of services, researchers and clinicians were recruited to form state‐based Advisory Groups. These groups were formed in co‐operation with the services approached in the initial consultations. Members included CEOs, nationally recognised researchers, medical clinicians, Men's group leaders, project officers and Social and Emotional Wellbeing (SEWB) staff. A list of the Advisory Groups is in Appendix 1.

### 2.3 Workshops

Following initial consultations, stakeholders were invited to participate in 3–4 h workshops via an electronic SMS4DeadlyDads flyer and invitation distributed through service contacts formed during the initial consultations in each state. Representatives from mainstream perinatal services in contact with Aboriginal and Torres Strait Islander communities and from Aboriginal and Torres Strait Islander services were invited. Participation was voluntary, and no payments were offered for travel or attendance.

Following introductions, the SMS4DeadlyDads service was described, and the purpose of the workshop was explained. Existing support for fathers was discussed with presentations from local men's groups. Participants self‐selected into small groups to consider examples of the draft messages. Evaluations involved stickers to denote acceptable, good and problematic messages with comments and suggested improvements. After each workshop draft, messages were edited and, after checking with the Advisory Group, presented to the following workshop. An outline of the workshop has been added as Appendix 2.

In addition to workshops, meetings were held with perinatal services supporting Aboriginal and Torres Strait families to discuss the messages and how they might be incorporated into the clinical work with mothers and families. The Advisory Groups in each state reviewed the messages where further work was indicated, and the draft final set was then reviewed by Red Nose staff to advise on the order in which the messages should be sent.

## 3 Results

In this section, the understandings from the first meetings with community members are presented, followed by a description of the steps in drafting and evaluating the messages for grieving fathers. Then, the way that cultural safety figured in the discussions and message development is described.

Initial meetings were held in Queensland with representatives of Apunipima, Wuchopperen, Mookai Rosie, Torres and Cape HHS (Cairns), Mura Kosker, Cairns and Hinterland HHS (Thursday Island), in New South Wales with representatives of Awabakal and Miyay Birray (Newcastle and Moree) and in South Australia with representatives of the ICARE project developing culturally safe birthing services for women (https://sahmri.org.au/research-topics).

The initial discussion in each case focused on the provision of support for new fathers in general and in cases of pregnancy loss or neonatal death. In every discussion, staff from community organisations made similar points:

‘*There's a real need out there for fellas within our communities. Isolation is a big issue for our men’*.

‘*There's nothing in place in these situations for fathers…’*

‘*When there is a loss and dads are bereaved, they're really sad and they don't know who to reach out to. Usually mum is really upset obviously as well. So they might, you know, go and do things like get into fights or go and have a drink because they just don't know how to deal with it’.*

The frequency of deaths in the communities was also raised in urban and rural discussions. The need to avoid retraumatising community members was stressed, especially in smaller communities. An Indigenous medical officer returning from remote communities commented:

‘*Most of the time we're just getting over the last funeral, and the next one's already started. In some communities they can't even have kids birthday parties, because, you know, playing happy and stuff like that, but they never can, because there's always somebody dying. And these are small communities, not big ones. So, when somebody passes, everybody knows them and everybody misses them, you know. It's a different kind of thing sometimes to depression—the grief is quite different. It's kind of a pervasive, ongoing, sustained grief. I suppose it's no wonder, then, that we don't have a whole lot of dads rushing up us saying, “Let me tell you about what's happening with my miscarriage.” It's no wonder that it's not talked about so easily’.*

Hospital and community services described the way that arrangements for mothers coming to major hospitals to give birth, in some cases from great distances, meant that fathers were rarely able to be involved in the later stages of the pregnancy and the birth. In cases where accommodation was provided for mothers travelling from remote areas, accommodation for fathers accompanying the mothers was not possible, and when fathers did also travel down, the availability of alcohol frequently caused difficulties.

The availability of the SMS4DeadlyDads messages for Indigenous fathers was welcomed by all services and community members as ‘something for the men’, and the proposed development of messages for grieving fathers after pregnancy loss or stillbirth received strong support.

### 3.1 The Process of Message Development

An initial set of draft messages for grieving Aboriginal and Torres Strait fathers was adapted from those developed with mainstream fathers, mothers and staff from Red Nose, a national charity supporting grieving parents for delivery to mainstream fathers [8].

A sample of the adapted messages was presented at the December 2023 workshop in Cairns at Wuchopperen Health Service. The workshop was attended by representatives of 22 Aboriginal and Torres Strait services and 16 mainstream services. Comments from the groups were recorded by the group. Table 2 includes sample comments from Mainstream (M), Indigenous (I) and Mixed Indigenous and Mainstream (MG) groups. See Table 2 for examples of the evaluations.

**Table 2:** Examples of evaluation of draft messages, Wuchopperen workshop. Self‐selected groups were denoted I = Indigenous, MG = mixed Indigenous and non‐Indigenous and M = mainstream (non‐Indigenous).

4Dad: Here is info you can share with your family, friends and workmates. It will give them an idea of what you are going through and what they can do. [*StaticLink*]	I: Cross out family, friends and work mates. Which way Bro. Do you want some info to yarn with your mob. LINK…. I: Link for different indigenous translations M: after ‘friends and work mates’ add ‘if you would like to’, ‘if you choose to’
4Dad: Talk to family and friends who are close to you and who you respect, who will not judge you.	I: Which way Bro. Don't forget to reach out to your supports for yarn if you feel that will help MG: after ‘respect’ add ‘and trust’ MG: NW Replace ‘talk’ with ‘yarn’, ‘respect’ with ‘trust’, ‘who will not judge you’ with Yarning helps…’
4Dad: You are likely to be your partner's most important supporter. She needs to know you have got her back and that she is important to you. Remind her often.	MG: maybe mention ‘being a team’ otherwise this is great! I: Sticky note: Rub back. Ya right Bub' *Comment at table 'say he should rub her back and ask ‘Ya right Bub?* I: Which way Bro. Don't forget to check in with babies mother. She be feeling it too.
SMS4Deadlydads will send 15 weeks of messages with info and advice to help you navigate these difficult times. You can share some with your partner. You can opt out at any time by replying ‘STOP’.	I: Pictures more useful—visual practical. Too many words, needs validation. ‘Which way Bro. Thinking of you in this sorry time Losing a little one early can be very feeling isolated. Don't be frighten for reach out to your supports for yarning’ (Sad time, you might be like a Bala yarning). Suggestion voice message instead of SMS Black fella voice. *Comment at table ‘You can hear it (that he has had loss) in his voice’* MG: replace ‘15’ with ‘8’ replace ‘navigate’ with ‘go through’ M: replace ‘navigate’ with journey. Replace ‘you can share’ with ‘you can share or not share’. Sticky note: Acknowledge loss, support for dad and mum, voice to text, visual and animation, for example, GIF
4Dad: Parents sometimes feel guilty that they couldn't protect their baby. Guilt is a normal part of grief. If it's overwhelming, call Red Nose on 1300 308 307	I: Keep it simple ‘it's Ok to feel guilt’ rather than waiting til overwhelming ‘You can call Red Nose at anytime’ MG: Suggest service that is in their region ‘red nose may not be a service that is known’ I: Which way Bro. Its important you or baby mothers did nothing wrong. If you feel shame connect with supports in your area. LINK…
4Dad: Many dads try to keep it together while their partner grieves. Feel your grief and acknowledge it. It will help you cope while you care for those around you.	M: rather than ‘keep it together’ use ‘many dads feel they need to be strong’ ‘it's Ok to reach out for support’ I: Which way Bro. If you lose little one early it is important to grieve your way and stay in touch withyour supports. Grieve together.
4Dad: If one wanted to try for another pregnancy whilst the other one wants time to think about it. Wait until you're both ready. It can help your relationship stay on track.	MG: ‘Keep communication open + honest make sure you're both ready for another baby—there's no right time’ ‘When you are ready it's a good time to see your Health Care provider’ I: Culturally inappropriate—mob business not SMS M: Grief is with an individual—this one has two grieving partners

These messages and others from the initial draft set were discussed at face‐to‐face workshops in Lake Macquarie and Newcastle NSW, Port Augusta and Adelaide SA and Mareeba and Cairns QLD. In addition, meetings were held with clinicians from mainstream and maternal health services and Aboriginal Community Controlled health services. The messages were also discussed at regular meetings of the three Advisory Groups.

The final set of messages, see examples in Table 3, involved contributions from a total of 139 Aboriginal and Torres Strait Islander service representatives and 89 mainstream service representatives. In addition, numerous representatives and the Advisory Groups discussed the resources to be linked to the brief messages. The staff at Red Nose reviewed the messages and advised on the order to be followed in delivering them.

**Table 3:** Examples of final messages.

Week	Message
1	Hey brother, we're sorry to hear things didn't work out with your little one. Losing a bub is tough. We hope these messages give some comfort and support in this sorry time. Some messages have links to more info if this is something you want.
1	Grief due to a loss can make it hard for mums and dads to think. Your partner might want you along at check‐ups to support her.
2	Counselling comes in different ways: through groups, online, over the phone, one‐on‐one and family counselling. This link has info about some options
2	It's tough to see the world move on when grief for your bub is intense and raw. This can be felt most strongly when going back to work or back to footy or other social settings. Here are some positive ways to help you stay strong.
3	It's hard to hear someone you love talking about their grief without wanting to fix it. Maybe just being there is enough even without words.
3	Yarning up with some elders can be healing too. They might have stories about bub passing
4	Physical changes after a pregnancy loss can be sad reminders for mums. You can find out more about what you might be able to do to help: Link
4	Our mob reacts to stress in different ways. We can get angry, get quiet, self‐medicate…. It's important we deal with stress in healthy ways. Here are some ideas
5	Most mums want to know their partner is feeling the loss too. Shared journeys, even hard ones, can make relationships stronger
5	Here are some ideas for how to tell people you haven't seen about your loss. ‘Hey Bro can you let the mob know Bub didn't make it. Can I call you later for a yarn. PS don't post on social media’ ‘Hey Bro. We lost our little one. She didn't make it I'm hurting real bad. I'm going OK but it's made me real sad. Can you let the mob know. Can you call me in a while I might want to yarn then’ (Tip: Ring someone close to deliver the message)

The messages span several domains, with an emphasis on father self‐care and father–partner relationships. Fathers' self‐care messages highlighted emotional well‐being through acknowledging emotions, seeking help, taking leave, sharing conversations and managing expectations (at the family–workplace–childrearing nexus). Father–partner texts included supporting the partner's emotions, fostering partnership, healing together, managing practical tasks and planning for future pregnancy.

### 3.2 Navigating Cultural Safety While Engaging With Communities

In the visits by team members to locations across the three states, two levels of discussion were apparent. The first question ‘What is available for young fathers in this area?’ brought an immediate response, attesting to the lack of services for this group and their high level of needs. In several locations, ‘Men's Groups’ had been initiated and then terminated when funding expired. The availability of a culturally‐specific text messaging service, which was already available to support local fathers, provided a positive, solution‐focused tone to the conversations.

When the topic of stillbirths was raised, the conversations were more cautious as community participants and service providers gathered accounts of how these losses were managed. There was no uniform approach evident in these discussions. In many communities, only the immediate family would come together to mark the passing of the baby. The notion of a sorry business, involving many family members, participating in extensive ceremonies, was not applicable. In contrast, for those deaths occurring in the Torres Strait, cultural practices specified which family members could attend and the type of actions to be undertaken during ceremonies.

‘*At the hospital or at home they will tell them to contact the in‐laws, I can't touch in‐laws or look them in the eye but we be different up there we do say name. When there is a death in laws come to the front of the house. They don't come in all the family knows that there is a death. They don't ask. When they all together the head in law comes and he tells them the name of who died and that they are in a happy place. The in laws take care of the family to make sure that they grieve’.*

In many cases, it was emphasised that there is no widespread discussion of stillbirth in the community.

In most discussions, cultural directions relating to men's business and women's business were not raised as prohibitions in developing the support for grieving fathers and the situation of perinatal loss was accepted as ‘family business’. However, in discussion with elders from central Australia, the approach of engaging fathers during the pregnancy and birth was not supported.

At a meeting with 20 central Australian community members, a female elder stopped the discussion of men's roles during pregnancy and birth. All the men were ordered to leave, and the women talked. When the men were invited back into the room, they were told that this was ‘*wrong way* … *men don't go the hospital don't go with her … that sacred women's business’*. A similar point was raised at one of the workshops (see Table 2). However, it was acknowledged that in these communities, there was variation in how services and community members applied these strictures.

The sensitivity required to raise the issue with fathers who had a loss was reinforced in several ways. In most settings, community representatives had either experienced a pregnancy loss or stillbirth or knew of those who had. But few fathers, when contacted, were prepared to speak publicly about their loss, sometimes deferring appointments to discuss their experiences, explaining that they ‘were not ready’ to talk about these painful events. When fathers who had experienced loss did speak in the meetings, they were overwhelmed with emotions even when describing events many years in the past. The privacy of the text messaging service was seen as particularly useful in reaching out to grieving fathers and offering them support.

A senior staff member from an Aboriginal health service explained:

‘*[the SMS service] can be a place where can ask questions without feeling shame…. Maybe they might think they have silly question—but if it's goin' to somebody they don't know and it comes back then that's OK. They don't have to feel shame. No‐one's looking over their shoulder saying, “Oh, you should already know that” or “how come you don't know that?” So, the text service can be a safe place to get information’*.

## 4 Discussion

Consultation with Aboriginal and Torres Strait Islander communities is accepted as essential for designing culturally safe health resources, and national guidelines for ethical research have been developed. However, current guidelines do not specify the procedures to be adopted, and there is considerable variation in the consultation methods reported in the literature [24, 25]. This study reports on an iterative process of consultation with Aboriginal and Torres Strait Islander services, mainstream services supporting Aboriginal and Torres Strait Islander families and members of the community in three states.

The need for cultural safety was central to the process of consultation due to the nature of the project's aim: addressing fathers' grieving after a pregnancy loss or stillbirth. Apart from the obvious sensitivity attached to the loss of a pregnancy or the failure of the newborn to survive, cultural contexts of sorry business, men's and women's business were important factors to consider. Building trust between those taking part in the consultation and the research team was a critical step if a genuine contribution to the messages of support for grieving fathers was to be gained [26, 27].

In this study, the reputation, knowledge and extensive prior engagement of the cultural lead, Dr Mick Adams, facilitated the contact with Indigenous services and enabled ongoing commitment to the project aims. Similarly, members of the three Advisory Groups provided local contacts and perspectives, which assisted with providing a culturally safe environment for consultations and ensured that regional cultural differences were respected. A third factor aiding the engagement of community members and service providers was the immediate availability of the SMS4DeadlyDads message service for new fathers, as evidence of the capacity of the research team to supply tangible support to local men in the transition to fatherhood.

The nature of the messaging service being proposed also influenced the consultation process. The texts to be developed were pre‐defined as brief conversational statements with links to further information and suggestions for action. The participation requested of community and service staff was to supply advice, review drafts of messages, and, once the messages were available, alert new fathers within their catchment group to this new support service. No significant change in the existing services was implied in the collaboration request; however, the lack of referral points for fathers to be linked to the messages was recognised in every region.

Raising awareness of stillbirth in Aboriginal and Torres Strait Islander communities may require considerable promotion to establish widespread discussion in the community about perinatal loss. While sorry business cultural practices involving a wide circle of community members over days or weeks are assumed to be universal [28] in our discussions with communities in Queensland, New South Wales and South Australia, grieving activities following perinatal loss were often described as restricted to the immediate family. An extensive consultation involving face‐to‐face meetings with 18 communities in Queensland, Western Australia, Victoria, New South Wales and South Australia found that ‘Stillbirth/Sorry Business Babies is a difficult topic and is generally understood to be a taboo subject to talk about’ [7, p. 9].

In some locations, SEWB programmes were seen as suitable vehicles for raising the issue of fathers' distress following pregnancy loss or stillbirth. The principles underpinning SEWB programmes, including a strengths‐based approach, and emphasising connection to community, country, family and spirit, align with the discussion of suitable messaging for distressed and grieving fathers [29]. As many Aboriginal Community Controlled Organisations offer men's groups as part of their SEWB programmes, there is potential for raising awareness of stillbirth and its impact on fathers in these groups [13].

The unforeseen loss of a pregnancy or the shock of a stillbirth heightens the risk to the father's identity, and he may lose connection with family and himself. In this regard, the medium of text messaging was seen as advantageous, as the texts can be read at any time and in private and do not require disclosing painful events to others. However, it was recognised that texts alone would not constitute sufficient support for fathers. More informal and locally based services are needed. Although telephone support is available through the Aboriginal and Torres Strait Islander national crisis support line 13YARN, it was evident that not all community members would find this acceptable. Comments in discussions asserted a preference for ‘someone they know’ locally rather than an anonymous call centre. The need for more services in Indigenous communities is an ongoing issue, not restricted to perinatal loss. However, the lens of stillbirth, with its undoubted impact on a father's well‐being, highlights the lack of attention to fathers across the perinatal service sector, not only in local service capacity but in policy and guidelines which shape the health system surrounding pregnancy and birth.

### 4.1 Implications

This study has implications for the prevention, treatment and care surrounding stillbirths occurring in Aboriginal and Torres Strait Islander communities. Efforts to reduce stillbirth rates in mainstream Australia have focused exclusively on mothers. The Still Six Lives [30] national stillbirth public awareness campaign targeted only mothers, and clinical care guidelines are addressed exclusively to the mother as a patient of the health service [31]. Father's role in assisting with stillbirth prevention behaviours and the treatment and care of fathers following a stillbirth are not considered. Materials for Aboriginal and Torres Strait Islander communities such as Stronger Bubba Born [32] are modelled on mainstream campaigns and while family and community are mentioned, only the mother is addressed. The failure of national guidelines and campaigns to incorporate Indigenous contexts and culturally appropriate models of care has been recognised [7]; however, the absence of attention to Aboriginal and Torres Strait Islander fathers has not been examined. The invisibility of fathers in the policy and guidelines for care after a stillbirth is remarkable when the need is highlighted by services supporting grieving families, and the cost for depressed fathers can be devastating. As a submission from Grief Australia into the South Australian inquiry into stillbirth stated, ‘Healthcare systems have a focus on providing immediate support to mothers when they experience stillbirths. However, there is limited or, in some instances, no care provided to grieving fathers even though they are experiencing profound grief associated with losing a baby’ [4]. The consequences of isolation and disconnection from support for Aboriginal and Torres Strait Islander males can be seen in the national statistics on self‐harm and suicide [33]. Once the messages are made available through SMS4Deadlydads and through the Red Nose website, the take‐up of this form of support will be apparent. It is hoped that the existence of this resource will highlight the need for attention to fathers' needs and stimulate policymakers and services to review policy and procedures to more effectively support families by supporting the fathers.

The HTC research project will finally be tested once the text messages are delivered to grieving fathers. However, the production of messages through collaborative co‐design across three states is in itself an achievement. Having a positive digital resource (available everywhere) to offer communities and research leaders with many years of experience working with Aboriginal and Torres Strait Islander men was a key feature of this successful approach.

### 4.2 Strengths and Limitations

Strengths of this study included framing the message development as a practical support for grieving fathers, which resonated with service providers who were acutely aware of the lack of programmes for fathers. Having experienced and well‐known Indigenous men as leaders in initiating discussions facilitated the engagement of community members and service staff. There were also limitations evident in the process of developing messages. While the areas visited included urban, rural and remote areas, the visits were restricted to three states, and only Thursday Island was visited in the Torres Strait. Cultural differences and perspectives from a wider range of communities would be an advantage in broadening the content of the texts. The procedure, which focused on brief messages for fathers affected by stillbirth, constrained the discussions of the issues surrounding Aboriginal and Torres Strait Islander fathers and highly relevant topics, such as imprisonment, which were flagged in several discussions, were not pursued.

## 5 Conclusions

The consultation process described in this study is unique in its specific focus on the well‐being of Aboriginal and Torres Strait Islander fathers grieving a pregnancy loss or stillbirth. Aboriginal‐controlled and stakeholder services, as well as community members, recognise the gap in support for grieving fathers and the lack of programmes targeting young men as they transition to fatherhood. However, the ‘taboo’ surrounding stillbirth and the mother‐focus of perinatal services limited the depth of discussion and required revisiting locations and topics to allow time for debate to occur within communities on the message content and cultural safety aspects. The existence of this resource may highlight the lack of attention to Aboriginal and Torres Strait Islander fathers' needs and stimulate policy makers and services to review policy and services to more effectively support families by supporting the fathers.

## Author Contributions

**Craig Hammond:** conceptualisation, methodology, writing – review and edit, data curation, project leadership. **Mick Adams:** conceptualisation, methodology, writing – review and edit, data curation, project leadership. **Richard Fletcher:** writing – original draft, writing – review and edit, data curation.

## Ethics Statement

Ethical approval was received from the University of Newcastle Human Research Ethics Committee (H‐2022‐0016) and from the Aboriginal Health and Medical Research Council (1874/21).

## Consent

All Indigenous services and stakeholders agreed/consented to take part in the consultation process.

## Conflicts of Interest

The authors declare no conflicts of interest.

## Supporting information

Appendix 1 Advisory Groups.

Appendix 1 Advisory Groups.docx.

## Data Availability

Data available from the corresponding author upon reasonable request.
