# Supplementary material for: Consulting With First Nations Communities to Develop Text‐Based Support for Grieving Fathers
Source: Health Expect. 2025 Sep 29;28(5):e70450. doi: 10.1111/hex.70450 (PMC12477625; doi:10.1111/hex.70450)
Supplement: Supplementary file 1 — Appendix 1 Advisory Groups. [file HEX-28-e70450-s001.docx]

Appendix 1 Healing Through Community SMS4DeadlyDads Advisory Groups

| **Surname** | **First Name** | **Aboriginal/ Torres Starit Islander** | **Organisation** | **Group** |
| --- | --- | --- | --- | --- |
| Bernard | David | Yes | Apunipima | QLD |
| Roderick | Thompson | Yes | Mulungu Aboriginal Corporation Primary Health Care Service | QLD |
| Lyndon | Reilly | Yes | University of NSW | QLD |
| Philip Uel | Bani | Yes | Wuchopperen | QLD |
| Mark | Wenitong | Yes | National Mental Health Commissioner | QLD |
| Marsat | Ketchell | Yes | QLD Health | QLD |
| Jake | MacDonald | Yes | University of Newcastle | NSW |
| Jason | Smith | Yes | Awabakal, CEO | NSW |
| Rodney | Smith | Yes | Awabakal | NSW |
| Paul | Mason | Yes | Probation & Parole | NSW |
| Ian | Eggins | Yes | Wandiyali | NSW |
| Barry | McGrady | Yes | Allambi Care | NSW |
| Eugene | Warrior | Yes | SA Health | SA |
| Dylan | McKenzie | Yes | Marnbi | SA |
| Clinton | Bennell | Yes | Nunga Mi:Minar | SA |
| Douglas | Clinch | Yes | SA Health | SA |
| Alwin | Chong | Yes | Arneychong consulting | SA |
