# Supplementary material for: Consulting With First Nations Communities to Develop Text‐Based Support for Grieving Fathers
Source: Health Expect. 2025 Sep 29;28(5):e70450. doi: 10.1111/hex.70450 (PMC12477625; doi:10.1111/hex.70450)
Supplement: Supplementary file 2 — Appendix 1 Advisory Groups.docx. [file HEX-28-e70450-s002.docx]

Appendix 2 Workshop Outline

The Healing Through Community (HTC) Grieving Fathers Project

WORKSHOP OUTLINE

1. A ‘Welcome to Country’ from an Aboriginal or Torres Strait Islander person with authority to speak for the people whose land we were meeting on (land never ceded) or, an Acknowledgement of Country by a senior member of the community commenced the workshop.
2. SMS4DeadlyDads presenters, service, and community representatives’ introductions
3. Workshop outline and safety permission (leave whenever necessary if distressed)
4. Presentation (including video material featuring Indigenous fathers) by SMS4DeadlyDads staff on the reasons for this workshop, the activities planned and the expected outcomes.
5. Presentations by local community members on existing (or recently closed due to lack of funding) men’s groups or programs for men.
6. Explanation of the draft text messaging examples (usually distributed on A3 size sheets of paper) and the task: to code the texts (good, OK or problematic) and write comments.
7. Participants self-select into small groups to discuss the texts.
8. SMS4DeadlyDads staff circulate between groups clarifying the tasks or the service features
9. After a period of 45 -60 minutes the sheets are collected and a final discussion with the whole group covered the experience of rating the texts, issues raised during the task and suggestions for future father-focused support.
10. Lunch is provided either before or after the workshop
